# Supplementary material for: Association between oral microbiome and seven types of cancers in East Asian population: a two-sample Mendelian randomization analysis
Source: Front Mol Biosci. 2023 Nov 21;10:1327893. doi: 10.3389/fmolb.2023.1327893 (PMC10702768; doi:10.3389/fmolb.2023.1327893)
Supplement: Supplementary file 1 [file DataSheet1.ZIP › Supplementary Materials/MR plots of saliva/saliva═╝/Prostate cancer/pheno.1398_to_prostate cancer_leave_one_out.pdf]

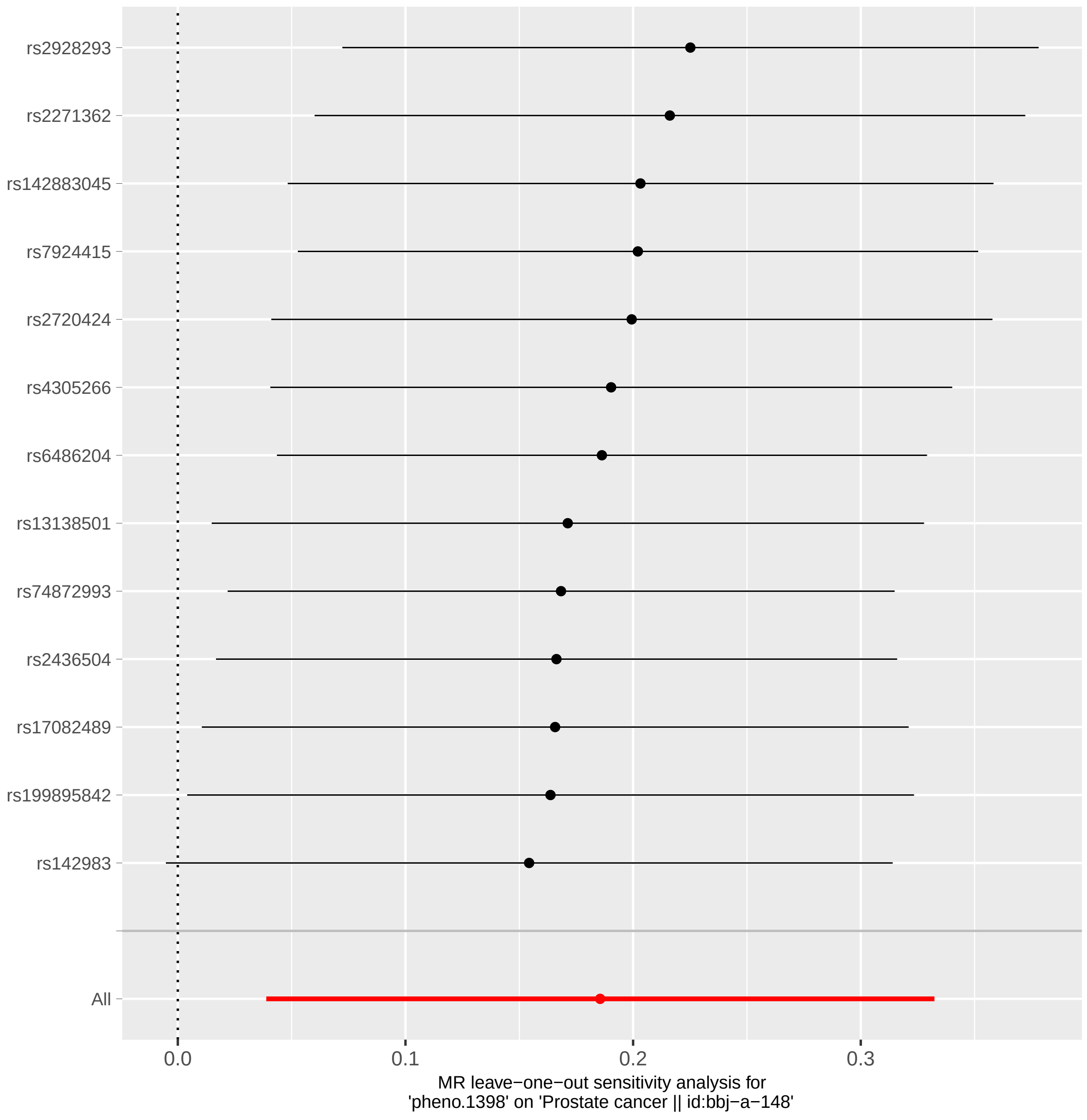

rs2928293
rs2271362
rs142883045
rs7924415
rs2720424
rs4305266
rs6486204
rs13138501
rs74872993
rs2436504
rs17082489
rs199895842
rs142983
All
0.0
0.1
0.2
0.3
MR leave−one−out sensitivity analysis for
'pheno.1398' on 'Prostate cancer || id:bbj−a−148'
